# A Question of Eugenics

**Published:** 1920-06-19

**Authors:** 


### A Question of Eugenics.

The difficulties and importance of the proper care of mentally defective children formed the subject of several well-informed addresses at the meeting of the West Lancashire Association for the Care of the Mentally Defective. Dr. E. Norwood East, Medical Officer of Walton Prison, and Dr. C. J. Macalister were the principal speakers, and their experiences emphasised the seriousness of the social menace in the number of mental defectives. They commit all kinds of crime, the only one whereof Dr. East has been unable to find a definite record being that of treason. Once the habit of crime becomes firmly rooted it is difficult to eradicate, and the prevention of crime in this respect is itself an enormous field for social workers. The trouble is—and here is a pathetic side of the matter—that parents entertain hope against hope that at some definite period there may be a sudden return to sanity, and, with a solicitude that is marked in relation to mentally defective children, they cling to them in this anxious expectation. But it is mostly the case that under scientific care the children would have better opportunities of improvement, and it is amazing how children who have been regarded as deficient improve mentally when deformities are removed. Discipline seems also to be most effective, and Dr. Macalister says many defectives joined the Army and did good work, but failed to do any work afterwards, and there are a considerable number of such cases which could be made use of under discipline, where they have no need to exercise any initiative.

Fundamentally, it is a question of eugenics, but existing defectives ought certainly to be safeguarded against themselves and against others, and efforts continued to make them as useful and as harmless as possible to the community. It requires, however, something more than the exercise

**THE PUBLIC WELL-BEING**—(*continued*).
of unaided public opinion to tackle, for example, the question of the production of children by feeble-minded people, which seems to be the elementary starting-point of prevention, and, following that, the question of the production of children by diseased parents. Unfortunately, public opinion is more inclined to be bored with the general discussion of mental defectives than to be stirred to action. It can work itself up into great excitement over some remote foreign political relationship, but remain perfectly placid to social menaces on the doorstep—that is, if they are not political or industrial.

